# Offspring Birth Weight Is Associated with Specific Preconception Maternal Food Group Intake: Data from a Linked Population-Based Birth Cohort

**DOI:** 10.3390/nu12103172

**Published:** 2020-10-16

**Authors:** Nastaran Salavati, Petra C. Vinke, Fraser Lewis, Marian K. Bakker, Jan Jaap H.M. Erwich, Eline M. van der Beek

**Affiliations:** 1 Department of Obstetrics and Gynecology, University Medical Centre of Groningen, University of Groningen, 9700 AB Groningen, The Netherlands; m.k.bakker@umcg.nl (M.K.B.); j.j.h.m.erwich@umcg.nl (J.J.H.M.E.); 2 Department of Epidemiology, University Medical Centre of Groningen, University of Groningen, 9700 AB Groningen, The Netherlands; p.c.vinke@umcg.nl; 3 Danone Nutricia Research, 3584 CT Utrecht, The Netherlands; fraser.lewis@nutricia.com (F.L.); e.m.van.der.beek@umcg.nl (E.M.v.d.B.); 4 Department of Pediatrics, University Medical Centre of Groningen, University of Groningen, 9700 AB Groningen, The Netherlands

**Keywords:** preconception, dietary intake, food groups, birth weight, birth cohort

## Abstract

The preconception period has been recognized as one of the earliest sensitive windows for human development. Maternal dietary intake during this period may influence the oocyte quality, as well as placenta and early embryonic development during the first trimester of pregnancy. Previous studies have found associations between macronutrient intake during preconception and pregnancy outcomes. However, as food products consist of multiple macro- and micronutrients, it is difficult to relate this to dietary intake behavior. Therefore, the aim of this study was to investigate the association between intake of specific food groups during the preconception period with birth weight, using data from the Perined-Lifelines linked birth cohort. The Perined-Lifelines birth cohort consists of women who delivered a live-born infant at term after being enrolled in a large population-based cohort study (The Lifelines Cohort). Information on birth outcome was obtained by linkage to the Dutch perinatal registry (Perined). In total, we included 1698 women with data available on birth weight of the offspring and reliable detailed information on dietary intake using a semi-quantitative food frequency questionnaire obtained before pregnancy. Based on the 2015 Dutch Dietary Guidelines and recent literature 22 food groups were formulated. Birth weight was converted into gestational age-adjusted z-scores. Multivariable linear regression was performed, adjusted for intake of other food groups and covariates (maternal BMI, maternal age, smoking, alcohol, education level, urbanization level, parity, sex of newborn, ethnicity). Linear regression analysis, adjusted for covariates and intake of energy (in kcal) (adjusted z score [95% CI], P) showed that intake of food groups “artificially sweetened products” and “vegetables” was associated with increased birth weight (resp. (*β* = 0.001 [95% CI 0.000 to 0.001, *p* = 0.002]), (*β* = 0.002 [95% CI 0.000 to 0.003, *p* = 0.03])). Intake of food group “eggs” was associated with decreased birth weight (*β* = −0.093 [95% CI −0.174 to −0.013, *p* = 0.02]). Intake in food groups was expressed in 10 g per 1000 kcal to be able to draw conclusions on clinical relevance given the bigger portion size of the food groups. In particular, preconception intake of “artificially sweetened products” was shown to be associated with increased birth weight. Artificial sweeteners were introduced into our diets with the intention to reduce caloric intake and normalize blood glucose levels, without compromising on the preference for sweet food products. Our findings highlight the need to better understand how artificial sweeteners may affect the metabolism of the mother and her offspring already from preconception onwards.

## 1. Introduction

The first 1000 days of life are known as a critical window for the effect of environmental influences (including maternal dietary intake) [1] on early growth and development and has become a popular domain for researchers and health care professionals. Within this window, much of the focus has been on the infant before and after birth and to a much lesser degree on women during their preconception period, despite the acknowledgment of the importance of the preconception phase on pregnancy and later health outcomes for both mother and child. When women are in good health and nutritional status before pregnancy, this beneficially influences the health of the child in two ways. First, nutrition may already influence the quality of the oocyte and its environment before conception [2]. Second, after conception, the first trimester of pregnancy is the period when most fetal organs and the placenta are formed, and when many women are not yet aware of being pregnant. If women are in an adequate nutritional status before pregnancy, this may have beneficial effects mainly during this first, important, part of pregnancy.

Previous studies have investigated the associations between macronutrient intake and birth weight [3,4]. Although results from these analyses may provide useful insights to generate hypotheses regarding nutrient intake and outcomes, in practice we do not consume isolated macronutrients. Consequently, multiple food groups may contribute to the observed associations and it has indeed been found that food-based scores may have stronger associations with chronic diseases [5]. The effects of foods likely reflect complex synergistic contributions from and interactions among food structure, preparation methods, macronutrient quality (e.g., glycemic index and fiber content in case of carbohydrates), and micronutrients content [5]. In addition, as people modify their nutrient intake primarily by their choice of foods, dietary recommendations are generally based on epidemiologic analysis with foods (e.g., food items or groups), as opposed to analysis based on nutrient intake [6]. Therefore, our objective is to further explore the association between intake of food groups and pregnancy outcome, more specifically the birth weight of the offspring.

We investigate the association of 22 food groups, classified based on the food-based 2015 Dutch Dietary Guidelines [7,8], and birth weight in the Perined-Lifelines linked birth cohort [9]. Subsequently, we examined which macronutrients may contribute to the association found between food groups and birth weight.

## 2. Materials and Methods

This study was part of the Perined-Lifelines linked birth cohort, a cohort established by linking the Dutch national birth registry (Perined, [10]) and the population-based Lifelines Cohort Study [11]. The Lifelines Cohort study was conducted according to the principle of the Declaration of Helsinki and is in accordance with the research code of the University Medical Center Groningen, The Netherlands (METc number: 2018/506). The linking of data from Lifelines with other existing databases was covered by the informed consent filled in by the participants.

### 2.1. Overview of the Perined-Lifelines Linked Birth Cohort

The Perined-Lifelines linked birth cohort was created by linking two existing databases; a large population-based cohort study (The Lifelines Cohort study, [11]) and the national birth registry (Perined, [10]), through a “trusted third party” (‘ZorgTTP’ Houten, The Netherlands), facilitated by Mondriaan project (UMCG)/Lygature (Utrecht, The Netherlands). Lifelines is a multi-disciplinary prospective population-based cohort study examining in a unique three-generation design the health and health-related behaviors of 167,729 persons living in the north of The Netherlands. It employs a broad range of investigative procedures in assessing the biomedical, socio-demographic, behavioral, physical, and psychological factors which contribute to the health and disease of the general population, with a special focus on multi-morbidity and complex genetics. The Perined-Lifelines linked birth cohort has been described previously in detail [9]. In brief, female participants from the Lifelines Cohort study who indicated in their first or second follow-up questionnaire to have delivered a child since the previous questionnaire were selected. The information collected at baseline (e.g., demographical variables, detailed nutrient intake through a Food Frequency Questionnaire (FFQ)) was considered as the pre-conceptional information available for that specific pregnancy. Since the Lifelines Cohort Study does not collect information on pregnancy or pregnancy outcomes, the female participants from Lifelines were linked with the information on their pregnancy outcomes available via the national birth registry (Perined). This was done via corresponding pseudonyms in Lifelines and Perined, which was created based on three personal linking variables (birth date and 4-digits ZIP code of the residential address of the female participants from Lifelines, and birth date of their child). This resulted in a Perined-Lifelines linked birth cohort, containing information on dietary intake during the period prior to conception as well as pregnancy outcomes.

### 2.2. Study Group

Among the women in the Perined-Lifelines linked pregnancy cohort, the inclusion criteria for the present analyses were delivery of a live-born baby at term (≥37 weeks’ gestational age) and availability of information on birth weight of their offspring. Women with unreliable data for dietary intake were excluded from the analyses. Reliability of reported dietary intake was checked using the Goldberg cut-off method, which relies on the ratio of reported energy intake and basal metabolic rate [12,13]; a ratio below 0.50 or above 2.75 was considered as not reliable. Additionally, women with an intake of less than 500 kcal/day were considered as unreliable reported dietary intake [14,15], and therefore excluded for further analysis.

### 2.3. Dietary Assessment: Food Groups

Based on evidence underlying the 2015 Dutch Dietary Guidelines, Vinke et al. [16] categorized the 110 items of the FFQ used in the Lifelines Cohort into 22 food groups and labeled them as having a positive, negative, neutral, or unknown relation with major chronic diseases [7,8]. Nine positive groups (vegetables, fruit, whole grain products, legumes and nuts, fish, oils and soft margarines, unsweetened dairy, coffee, and tea), one neutral food group (eggs), three negative groups (red and processed meat, butter and hard margarines, and sugar-sweetened beverages) and nine unknown groups for which evidence is either absent or weak (potatoes, refined grain products, white unprocessed meat, cheese, savory and ready products, sugary products, soups, sweetened dairy, artificially sweetened products) were identified [8]. The same categorization of food groups was used within the current study. To represent relative food group intake, taking into account differences in energy intake between individuals, intake of the food groups was expressed in grams per 1000 kilocalories (kcal) instead of grams/day. For linear regression analysis, intake in food groups was expressed in 10 g per 1000 kcal to be able to draw conclusions on clinical relevance given the bigger portion size of the food groups. Based on recent literature [8], the nine positive and three negative food groups were combined into the Lifelines Diet Score (LLDS). For each food group (in grams/1000 kcal), intake was divided into quintiles to score an individual’s consumption compared to others in the study population. The quintiles range from 0 to 4, with 4 points being awarded to the highest quintile of consumption for positive food groups, and to the lowest quintile for negative food groups. The sum of the 12 component scores resulted in the LLDS, ranging from 0 to 48.

### 2.4. Maternal and Fetal Characteristics

Maternal age was defined as age at enrollment in Lifelines. Maternal education was assigned in three categories: low (no education, primary school, lower vocational or lower general secondary education), intermediate (intermediate vocational training or higher secondary education) and high (higher vocational or university) education. Maternal BMI was calculated based on measured height and weight at the Lifelines research sites at enrollment (baseline) to Lifelines. For the description of food group consumption within the cohort, stratified groups of BMI-quintiles were used. BMI quintiles were generated by use of the distribution within the study, whereby quintile 1 was defined as “low” BMI (lowest 20% in this cohort), quintiles 2 to 4 as “normal” BMI (middle 60% of this cohort and used as the reference) and quintile 5 as “high” BMI (highest 20% in this cohort). Maternal ethnicity was classified as either “white/European” and ”other.” Maternal alcohol use was divided into “alcohol use” (defined as alcohol use at moment of baseline/FFQ) and ”no alcohol use” [11]. Urbanization level was categorized as one, two or >/= three. Birth weight was recorded in grams in Perined and converted into a gestational age (GA)-adjusted z-score to adjust for variation of birth weight over gestational age.

### 2.5. Statistical Methods

Continuous variables were summarized by the median and IQR, and comparisons between groups were made by the Kendall’s tau rank correlation test. The associations between food group intake (exposure) and birth weight (outcome) were estimated by linear regression analyses. Maternal characteristics were considered as possible confounders based on a previous study within the Perined-Lifelines linked birth cohort [4]. Adjusted analyses were performed using multivariable linear regression analyses. Two multivariable linear regression models were performed for food groups that showed a significant association with birth weight to investigate which macronutrients within the food group might contribute to the association found. Model 1 included all main macronutrients (total protein, total carbohydrates, fat). Model 2 included nutrients quality (animal protein, plant protein, fat, mono-disaccharides, and polysaccharides). Statistical significance was assumed at *p* < 0.05. Analyses were performed in SPSS version 23 (IBM Corp., Armonk, NJ, USA).

## 3. Results

A total of 2368 women from The Lifelines Cohort Study were linked with their data in Perined. After excluding women who did not have reliable or missing dietary intake reported (resp. *n* = 427 and *n* = 168), pre-term births (gestational age <37 weeks; *n* = 110) and unknown sex of the child (*n* = 1), 1698 women remained available in the analysis. The characteristics of this group of women have been described in detail previously [4,9]. In short, the average maternal age was 29 years (25th–75th percentile: 27–32 years). Children were on average born at 39.4 weeks of gestational age with an had a mean birth weight of 3578 g (SD 472 g). Almost all women were of white (east/west European) ethnicity (97.8%), and the majority completed higher education (55.8%). The average maternal BMI was 23.8 kg/m^2^ (25th–75th percentile: 21.7–26.6), and average energy intake amounted to 1813 kcal/day (25th–75th percentile: 1545–2141). The average time between the FFQ (baseline) and the delivery of the child was 13 months (25th–75th percentile: 11–16 months). As described previously, there were no differences between the group of women with reliable vs. unreliable dietary intake that we consider to may influence our results in terms of selection bias.

### 3.1. Food Groups Intake in BMI Quintiles

The median consumption per food group in grams per 1000 kcal over groups of low (quintile 1), normal (quintile 2–4), and high (quintile 5) BMI is shown in Table 1. The intake of food groups legumes and nuts, tea, refined grain products, and sugary products decreased over the groups of BMI (resp. *p* = 0.003, *p* = 0.008, *p* = 0.03, *p* < 0.001) (Table 1). Intake of food groups eggs, red and processed meat, and artificially sweetened products increased over the BMI groups (Table 1) (resp. *p* = 0.05, *p* < 0.001 *p* < 0.001). The median LLDS in the complete cohort was 24 (IQR: 20–28) and decreased significantly over increasing BMI quintiles (*p* = 0.006).

### 3.2. Association of Intake of Food Groups and Birth Weight

First, unadjusted linear regression analysis was performed (data not shown); however, the R-squared of the models was very low and therefore not reliable (R-squared = 0.03). Adjusted linear regression analysis with adjustment for intake of energy (in kcal), maternal BMI, maternal age, smoking, alcohol, education level, urbanization level, parity, sex of the newborn, and maternal ethnicity showed an increase in explained variability of the model (R-squared = 0.15) and was therefore used for further interpretation of the analysis.

Adjusted linear regression analysis in the complete cohort showed that increased intake of food group artificially sweetened products was associated with increased birth weight (*β* = 0.001 [95% CI 0.000 to 0.001, *p* = 0.002]) (Table 2). To illustrate, per 10 g/1000 kcal intake of artificially sweetened products a day, birth weight is 0.001 SD higher (1SD = 472 g). As portion sizes of specific food groups are generally bigger than 10 g, this translates into a relevant further actual increased birth weight. To illustrate, a normal glass contains 200 g of, for example artificially sweetened beverages. So, with an intake of 2000 kcal per day, this makes 100 g/1000 kcal, resulting in an effect size of 10 × 0.001 = 0.01 SED = 4.7 g higher birth weight per portion artificially sweetened beverages.

In addition, increased intake of vegetables was associated with increased birth weight (*β* = 0.002 [95% CI 0.000 to 0.003, *p* = 0.03]) (Table 2). Increased intake of food group eggs was associated with decreased birth weight (*β* =−0.093 [95% CI −0.174 to −0.013, *p* = 0.02]) (Table 2). When linear regression analysis was performed in stratified groups of BMI quintiles, no association between food group intake and birth weight was shown.

### 3.3. Macronutrient Contribution to Associations between Food Groups and Birth Weight

To investigate which macronutrients from food group artificially sweetened products might contribute to the association found in linear regression analyses, we performed linear regression analysis with the macronutrients from this food group with birth weight. Analysis was performed twice; first only main macronutrients (total protein, fat, total carbohydrates), and second with the sub-macronutrients (nutrient quality) from animal and plant protein (instead of total protein) and mono- and disaccharides, and polysaccharides (instead of total carbohydrates) were included.

Total and animal protein from artificially sweetened products were significantly associated with increased birth weight (resp. (*β* = 0.047 [95% CI 0.004 to 0.089, *p* = 0.03]) and (*β* = 0.050 [95% CI 0.009 to 0.093, *p* = 0.02])) (Table 3). For analysis with macronutrients from food groups vegetables and eggs, we did not find any significant results.

## 4. Discussion

The aim of this study was to investigate the association of maternal diet based on specific food group intake during the preconception period with the birth weight of the offspring in a linked birth cohort of a representative group of women in the north of The Netherlands [4,9]. We showed that after correction for confounders, including maternal BMI and total energy intake, specifically higher intake of food groups “artificially sweetened products” and “vegetables” were associated with increased birth weight, and higher intake of food groups “eggs” was associated with decreased birth weight. In a previous study, we examined the association between preconception macronutrient intake and birth weight. The advantage of representing diet as specific compounds or group of compounds is that such information can be directly related to our dietary intake behavior. However, this approach does not provide any insights into what specific foods may contribute to the associations found.

Our finding that the food group artificially sweetened products was positively associated with birth weight is intriguing given recent studies carried out by researchers at Israel’s Weizmann Institute. They examined three artificial sweeteners (saccharin, sucralose, and aspartame) which are often incorporated into low-caloric snacks and beverages [17]. The research showed that all three sweeteners may induce metabolic changes such as glucose intolerance which is associated with an increased risk of diabetes mellitus and obesity. More specifically, they analyzed the possible associations between consumption of artificial sweeteners, microbiome composition, and metabolic outcomes in 381 of non-diabetic individuals (44% males and 56% females), and showed that intake of artificial sweeteners was not only associated with various clinical parameters such as BMI, blood pressure, HbA1 C%, and fasting glucose levels, but also with the presence of certain microbiota taxa [18]. These data suggest that artificial sweeteners and their associated microbiomes may play a crucial role in the regulation of glucose metabolism in humans. Artificial sweeteners stimulate intestinal sugar absorption [19], cause disruption of the ability of sweet taste to signal caloric consequences [20,21], they cause an increase in appetite [22], and finally, they elicit impaired glycemic or insulin responses [23].

The fact that we show a positive association between preconception intake of artificially sweetened products and birth weight may suggest that artificial sweeteners induce similar metabolic changes during preconception that may affect the offspring’s birth weight. During normal pregnancy, changes in insulin sensitivity are a normal physiological phenomenon that provides increased nutrient supply to the fetus. However, if insulin resistance develops and the beta-cell compensation of the pancreas is inadequate in secreting sufficient insulin to maintain normal glycemia in the mother, gestational diabetes mellitus may occur [24]. It would be interesting to further explore if and how the intake of products with artificial sweeteners may contribute to the pathophysiological mechanisms that also drive the association between gestational diabetes mellitus and obesity and increased birth weight [25].

We showed that the intake of artificially sweetened products increased over stratified groups of BMI; however, we adjusted for BMI via linear regression analyses, so our findings cannot fully be explained by the contribution of BMI. Unfortunately, we do not have information on gestational weight gain nor on glucose metabolism or occurrence of (gestational) diabetes mellitus available in this population, which could potentially confound the association with birth weight. However, the prevalence of diabetes mellitus (type 1 and 2) in the complete Lifelines Cohort Study is low, e.g., 4% [26], approximately 5% lower than reported by the WHO for the Netherlands [27]. We assume that the prevalence within the complete Lifelines Cohort Study is approximately the same as within our cohort of Lifelines participants, and therefore too low to have any major effect on the association found in this study [28]. In addition, as described in our previous paper, our cohort is relatively healthy in terms of BMI [4], one of the major risk factors for the development of gestational diabetes mellitus [29].

Within our study, the food group “artificially sweetened products” consists of two main food items. The first is “light soft drinks/lemonade without sugar,” and the second food item is “dairy drinks without sugar/with artificial sweeteners.” Given the fact that we found that it was (animal) protein within this food group that was positively associated with birth weight, this suggests that it is probably the food item ‘dairy drinks without sugar/with artificial sweeteners’ that is driving the positive association with birth weight as the other food item does not contain any protein. These results are in line with results from the Generation R study, a population-based prospective cohort study in Rotterdam, The Netherlands, who showed that higher intake of protein, especially animal protein, in early postnatal life was associated with a greater height, weight, and BMI in childhood up to 9 years of age in the offspring [30].

Besides “artificially sweetened products,” we also found an association between food groups “vegetables” and “eggs” with birth weight. Results from the Danish National Birth Cohort showed that fruit and vegetable consumption in pregnancy was positively associated with birth weight in well-nourished Danish women, especially among lean women [31]. The association between intake of vegetables and birth weight could potentially be due to the higher vitamins and minerals content of these foods, e.g., vitamin C and folic acid. In a study from Portsmouth, an increased birth weight was associated with vitamin C intake in early pregnancy [32]. Given the fact that the group of women in our cohort represents relatively healthy women with 60% of the women having a normal BMI according to the WHO classification [4,33], the association in our study may also be, partially, explained by improved micronutrient intake. As we did not have any information on micronutrient intake, we have not been able to further investigate this.

Regarding the association found between food groups, eggs, and birth weight, there is less scientific evidence to support this finding.

For many food groups (e.g., fruits, whole-grain products, red and processed meat) and overall diet quality (LLDS) we found a clear association with maternal BMI but most were not significantly associated with birth weight. More specifically we showed that mean diet quality (LLDS) slightly decreased with increasing BMI [8]. We also showed that on the one hand, sugar containing product intake decreased with increase of BMI, while on the other intake of artificially sweetened products increased. This shift in product preference with BMI has been described previously by several large-scale studies, including the National Health and Nutrition Examination Survey [34]. This supports our findings, shows our cohort to be representative, and suggests that similar findings can already be identified in much smaller but focused cohort studies like the Perined-Lifelines linked birth cohort.

As maternal BMI is a stronger predictor for birth weight than dietary intake [4], it is potentially the diet quality of the mother that can support a healthy pregnancy, but it is not something that is likely to have an acute effect on the offspring. Obviously, the diet may have already impacted maternal BMI, which is subsequently translated to the birth weight of their offspring. In this sense, maternal BMI may simply be the mediator between preconception dietary intake and birth weight.

Within our analysis we have not corrected for multiple testing given the more exploratory background of the analysis. Our results are indicating a direction of association between preconception dietary intake and birth weight. Further research should be performed within large cohorts investigating the association between dietary intake and birth weight to be able to draw more firm conclusions.

## 5. Conclusions

In summary, the present study provides information on food group intake and its association with birth weight in the linked Perined-Lifelines birth cohort. Our findings highlight the need to better understand how artificial sweeteners may affect the metabolism of mother and her offspring already from preconception onwards. Future research should be performed within large cohort studies to further investigate, confirm, and understand the associations found in the present study.

## Figures and Tables

**Table 1:** Median [p25–p75] consumption of the 22 food groups in the Perined-Lifelines linked birth cohort (*N* = 1698) in grams per 1000 kcal/day, stratified by BMI quintiles.

	Low BMI (Q1) ^1^ (20.3 [19.6–20.8]) *N* = 329 (100%)		Normal BMI (Q2-Q4) ^1^ (23.8 [22.6–25.5]) *N* = 1043 (100%)		High (Q5) ^1^ (30.5 [28.9–32.9]) *N* = 326 (100%)		*p* ^2^	Complete Cohort (*N* = 1698)	
LLDS ^3^	25	[21–29]	24	[20–28]	23	[19–27]	*0.006*	24	[20–28]
Energy (in kcal)									
Food groups									
Vegetables	50.6	[35.2–74.7]	54.7	[36.4–74.8]	53.5	[34.4–79.4]	0.51	53.8	[35.7–76.0]
Fruit	66.6	[37.6–106.8]	65.3	[29.6–109.7]	58.3	[28.4–113.4]	0.57	64.6	[30.5–109.8]
Whole grain products	53.9	[39.5–68.7]	52.5	[36.4–68.2]	50.8	[35.5–67.5]	0.31	52.1	[37.1–68.2]
Legumes and Nuts	7.9	[3.6–13.8]	6.3	[2.7–12.1]	5.8	[1.8–11.5]	*0.003*	6.4	[2.7–12.4]
Fish	4.8	[1.9–7.9]	5.3	[2.0–8.2]	5.5	[1.7–8.3]	0.51	5.3	[1.9–8.2]
Oils and soft margarines	8.4	[2.5–14.5]	8.7	[3.2–14.9]	8.6	[3.2–14.1]	0.52	8.7	[3.1–14.7]
Unsweetened dairy	65.0	[29.4–117.1]	71.8	[28.6–135.9]	82.3	[25.2–154.3]	0.14	71.3	[27.7–133.2]
Coffee	77.1	[0.0–175.6]	95.3	[0.0–204.0]	58.0	[0.0–191.4]	0.15	85.5	[0.0–194.6]
Tea	186.2	[104.2–290.9]	160.6	[73.3–282.8]	150.7	[61.9–269.7]	*0.008*	164.8	[77.6–282.1]
Eggs	3.6	[2.4–7.4]	4.0	[2.6–7.4]	4.3	[2.5–9.1]	*0.05*	4.0	[2.5–7.7]
Red and processed meat	29.5	[20.5–38.5]	33.9	[24.5–42.8]	35.9	[28.3–46.8]	*<0.001*	33.6	[24.6–43.0]
Butter and hard margarines	7.3	[2.4–13.5]	7.7	[2.2–14.5]	8.2	[2.4–14.4]	0.74	7.7	[2.3–14.4]
Sugar-sweetened beverages	61.0	[27.1–127.2]	65.7	[22.6–128.1]	65.0	[24.5–138.7]	0.94	64.7	[24.1–130.3]
Potatoes	28.5	[14.2–42.8]	29.5	[16.0–44.4]	27.7	[15.7–43.9]	0.84	28.9	[15.6–44.2]
Refined grain products	38.7	[27.2–56.2]	36.9	[25.1–53.1]	34.3	[24.6–49.4]	*0.03*	36.7	[25.4–53.0]
White, unprocessed meat	5.1	[2.9–8.1]	5.9	[3.7–9.0]	6.5	[4.5–9.9]	*<0.001*	5.9	[3.7–9.1]
Cheese	10.1	[5.7–17.3]	9.6	[5.0–16.7]	10.3	[6.1–17.0]	0.18	9.8	[5.4–16.9]
Savory and ready products	50.9	[35.8–69.0]	51.6	[37.5–67.4]	55.4	[40.6–73.2]	0.15	52.1	[37.6–69.3]
Sugary products	40.9	[30.1–51.8]	37.2	[27.1–49.1]	34.6	[23.4–45.6]	*<0.001*	37.4	[26.9–49.3]
Soups	15.1	[9.8–26.2]	15.9	[9.8–27.2]	16.3	[9.2–28.1]	0.77	15.9	[9.7–27.3]
Sweetened dairy products	47.0	[23.9–71.2]	47.7	[22.9–78.9]	48.6	[25.8–79.4]	0.29	47.7	[23.8–77.7]
Artificially sweetened products	12.9	[0.0–47.4]	21.5	[0.07–6.5]	43.9	[4.6–116.6]	*<0.001*	22.2	[0.0–77.4]

Data are median (IQR). ^1^ Q1 = Quintile 1 ranging from from 17.1–21.2 kg/m^2^, Q2–Q4 = Quintiles 2–4 ranging from 21.3–27.5 kg/m^2^, Q5 = Quintile 5 ranging from 27.6–47.3 kg/m^2^. ^2^ Two sided p value Kendall’s tau rank correlation test for continuous characteristics ^3^ LLDS = Lifelines Diet Score BMI = body mass index.

**Table 2:** Linear regression analyses of Lifelines Diet Score (LLDS) and food groups intake (in 10 g per 1000 kcal/day) in relation to birth weight (*n* = 1698, 100%).

	Adjusted Analysis ^1^		
Analysis	Coeff (95% CI) ^2^		*p*
LLDS ^3^	−6.4^E^−5	(−0.008 to 0.008)	0.99
Food groups			
Vegetables	0.002	(0.000 to 0.003)	0.03
Fruit	0.000	(−0.001 to 0.001)	0.73
Whole grain products	−0.001	(−0.004 to 0.002)	0.52
Legumes and Nuts	−0.003	(−0.009 to 0.004)	0.41
Fish	−0.001	(−0.009 to 0.007)	0.75
Oils and soft margarines	0.003	(−0.005 to 0.010)	0.48
Unsweetened dairy	0.000	(0.000 to 0.001)	0.22
Coffee	0.000	(−0.001 to 0.000)	0.14
Tea	6.26^E^−5	(0.000 to 0.000)	0.71
Eggs	−0.093	(−0.174 to −0.013)	0.02
Red and& processed meat	0.005	(−0.033 to 0.044)	0.78
Butter and hard margarines	0.045	(−0.031 to 0.121)	0.25
Sugar-sweetened beverages	−0.006	(−0.013 to 0.001)	0.08
Potatoes	0.000	(−0.025 to 0.026)	0.98
Refined grain products	0.000	(−0.027 to 0.027)	0.99
White, unprocessed meat	−0.033	(−0.132 to 0.067)	0.52
Cheese	−0.010	(−0.068 to 0.048)	0.73
Savory and ready products	−0.005	(−0.032 to 0.021)	0.70
Sugary products	0.014	(−0.026 to 0.054)	0.50
Soups	0.002	(−0.020 to 0.024)	0.83
Sweetened dairy products	−0.002	(−0.014 to 0.009)	0.68
Artificially sweetened products	0.001	(0.000 to 0.001)	0.002

^1^ Adjusted for intake of energy (in kcal), maternal BMI, maternal age, smoking, alcohol, education level, urbanization level, parity, sex of newborn, ethnicity, other 21 food groups intake. ^2^ Coefficients are expressed as z-scores, i.e., the unit for the coefficients is one standard deviation (SD). ^3^ Adjusted for intake of energy (in kcal), maternal BMI, maternal age, smoking, alcohol, education level, urbanization level, parity, sex of newborn, ethnicity.

**Table 3:** Linear regression analyses of macronutrients from food group “artificially sweetened products” (in grams per 1000kcal/day) in relation to birth weight (*n* = 1698, 100%).

	Adjusted Analysis ^1^		
	Coeff (95% CI) ^2^		*p*
Model 1-main macronutrients			
Total protein	0.047	(0.004 to 0.089)	0.03
Total fat	−0.003	(−0.034 to 0.027)	0.83
Total carbohydrates	−0.001	(−0.025 to 0.022)	0.93
Model 2-nutrient quality			
Animal protein	0.050	(0.009 to 0.093)	0.02
Plant protein ^3^	-	-	-
Total fat	−0.009	(−0.043 to 0.025)	0.59
Mono- and disaccharides	−0.004	(−0.027 to 0.020)	0.08
Polysaccharides	0.008	(−0.014 to 0.030)	0.48

^1^ Adjusted for intake of energy (in kcal), maternal BMI, maternal age, smoking, alcohol, education level, urbanization level, parity, sex of newborn, ethnicity, other food groups (21) intake. ^2^ Coefficients are expressed as z-scores, i.e., the unit for the coefficients is one standard deviation (SD). ^3^ No correlation between this macronutrient from this specific food group and birth weight within this model.
